# Hibernacula of bats in Mexico, the southernmost records of hibernation in North America

**DOI:** 10.1093/jmammal/gyae027

**Published:** 2024-05-03

**Authors:** Daniel Ramos-H., Ganesh Marín, Daniela Cafaggi, Cárol Sierra-Durán, Aarón Romero-Ruíz, Rodrigo A Medellín

**Affiliations:** Laboratorio de Ecología y Conservación de Vertebrados Terrestres, Instituto de Ecología, Universidad Nacional Autónoma de México, Coyoacán, Mexico City 04510, Mexico; Laboratorio de Ecología y Conservación de Vertebrados Terrestres, Instituto de Ecología, Universidad Nacional Autónoma de México, Coyoacán, Mexico City 04510, Mexico; School of Natural Resources and the Environment, University of Arizona, Tucson, AZ 85719, United States; Laboratorio de Ecología y Conservación de Vertebrados Terrestres, Instituto de Ecología, Universidad Nacional Autónoma de México, Coyoacán, Mexico City 04510, Mexico; Laboratorio de Ecología y Conservación de Vertebrados Terrestres, Instituto de Ecología, Universidad Nacional Autónoma de México, Coyoacán, Mexico City 04510, Mexico; Laboratorio de Ecología y Conservación de Vertebrados Terrestres, Instituto de Ecología, Universidad Nacional Autónoma de México, Coyoacán, Mexico City 04510, Mexico; Laboratorio de Ecología y Conservación de Vertebrados Terrestres, Instituto de Ecología, Universidad Nacional Autónoma de México, Coyoacán, Mexico City 04510, Mexico

**Keywords:** bat abundance, *Corynorhinus mexicanus*, *Corynorhinus townsendii*, hibernation, *Myotis velifer*, substrate temperature, torpor, abundancia de murciélagos, *Corynorhinus mexicanus*, *Corynorhinus townsendii*, hibernación, *Myotis velifer*, temperatura del sustrato, torpor

## Abstract

Although Mexico holds the southernmost hibernating bats in North America, information on winter behavior and hibernacula microclimate use of temperate Mexican bats is limited. We studied hibernating bats at high altitudes (>1,000 m a.s.l.) in northern and central Mexico during 5 consecutive winters. Our aims were to document and describe the hibernacula, winter behavior (such as abundance and roost pattern), and microclimates (estimated as adjacent substrate temperature) of cave-hibernating bats in Mexico. We found 78 hibernacula and 6,089 torpid bats of 10 vespertilionid species, increasing by over 50% the number of cave-hibernating bat species and quadrupling the number of hibernacula for Mexico. Hibernacula were at altitudes between 1,049 and 3,633 m a.s.l., located in 3 mountain ranges, mainly in oak and conifer forests. *Myotis velifer* was the most common species, followed by *Corynorhinus townsendii* and *C. mexicanus*. We recorded the adjacent substrate temperatures from 9 species totaling 1,106 torpid bats and found differences in microclimate use among the 3 most common species. In general, abundance of torpid bats in our region of study was similar to those in the western United States, with aggregations of tens to a few hundred individuals per cave, and was lower than in the eastern United States where a cave may hold thousands of individuals. Knowledge of bat hibernation is crucial for developing conservation and management strategies on current conditions while accommodating environmental changes and other threats such as emerging diseases.

## Teaser

Although bats hibernate across North America, very little is known about this phenomenon in Mexico. Here we increased by over 50% the number of known hibernating bat species and quadrupled the number of hibernacula for the country—and this is only the tip of the iceberg.

## Graphical Abstract

**Graphical Abstract:**
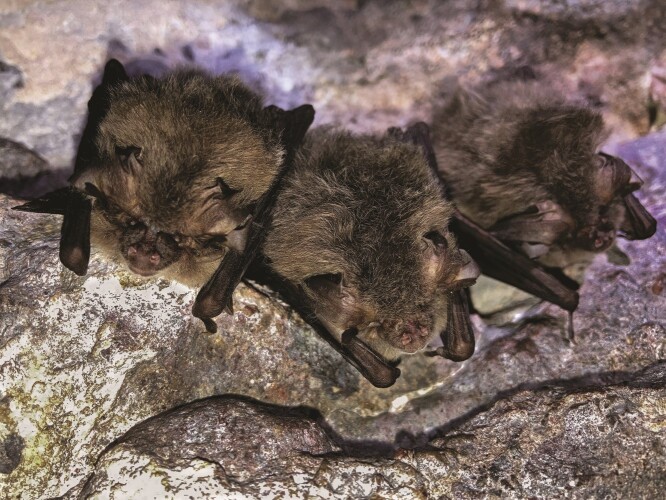

## Resumen

Aunque México tiene los murciélagos hibernantes más sureños en Norteamérica, la información sobre el comportamiento invernal y el uso de microclimas en los refugios de hibernación de los murciélagos templados mexicanos es limitada. Estudiamos a los murciélagos hibernantes en altitudes altas (>1000 msnm) en el norte y centro de México durante cinco inviernos consecutivos. Nuestros objetivos fueron documentar y describir las cuevas de hibernación, el comportamiento invernal (como la abundancia y patrón de percha), y el uso de microclimas (estimado como la temperatura del sustrato adyacente), de los murciélagos que hibernan en cuevas en México. Encontramos 78 cuevas de hibernación con 6089 murciélagos en torpor de 10 especies de vespertiliónidos, incrementando en más del 50% el número de especies de murciélagos que hibernan en cuevas y cuadriplicando el número de cuevas de hibernación para México. Las cuevas de hibernación estuvieron en elevaciones entre 1049 y 3633 msnm, localizadas en tres cadenas montañosas, principalmente en bosques de encinos y coníferas. *Myotis velifer* fue la especie más común, seguida por *Corynorhinus townsendii* y *C. mexicanus*. Reportamos las temperaturas del sustrato adyacente de 1106 murciélagos en torpor de nueve especies y encontramos diferencias en el uso de microclimas entre las tres especies más comunes. Aquí proveemos información relevante para especies de murciélagos templados en la ocurrencia más sureña de hibernación de murciélagos en Norteamérica. En general, la abundancia de murciélagos en torpor que encontramos fue similar a las del oeste de Estados Unidos, con agregaciones de decenas y algunos cientos de individuos por cueva; y fue menor que las del este de Estados Unidos, donde las cuevas pueden albergar miles de murciélagos. El conocimiento de la hibernación de murciélagos es crucial para el desarrollo de estrategias de conservación y manejo adecuadas en la actualidad y mientras se adaptan a los cambios ambientales y a otras amenazas tales como las enfermedades emergentes.

During cold periods or food shortages, temperate vespertilionid bats in North America can exhibit torpor (Brack 2007; Weller et al. 2018; Geiser 2021), which is a marked but controlled reduction of corporal temperature, metabolic rate, and other biological functions to conserve energy (Boyles et al. 2006; Altringham 2011; Geiser 2021). Depending on duration, torpor is typically labeled as daily torpor when it lasts less than 24 h or as hibernation when it lasts an extended period of days, weeks, or months (usually during winter), and requires utilization of stored body fat (Altringham 2011; Geiser 2021). Likewise, hibernating bats usually reduce minimum body temperatures to between 0 °C and 5 °C, but during daily torpor to between 17 °C and 26 °C (Geiser 2021). Hibernating bats typically use microclimates with high humidity and cold (above-freezing) stable temperatures (Altringham 2011; Perry 2013), but such conditions can vary among and within species along temporal and geographical gradients (Brack 2007; Altringham 2011; Geiser 2021). Factors that influence microsite selection operate at different levels and scales including latitude, altitude, vegetation, and hibernacula morphology (Dunbar and Brigham 2010; Perry 2013; Piksa et al. 2013; Meierhofer et al. 2019a), but low ambient temperatures are the main criteria for hibernation in temperate regions (Altringham 2011; Geiser 2021). The temperature of the substrate adjacent to torpid bats has been widely used to characterize microclimates (Brack 2007; Kim et al. 2013; Hopkins et al. 2021).

Mexico has 140 bat species (Medellín et al. 2008; Rivas-Camo et al. 2020), about 10% of the total species in the world, of which 8 vespertilionids are known to hibernate. Diversity patterns and distribution ranges of bats in Mexico are directly influenced by altitudinal variability (sea level to 5,848 m a.s.l.), complex topography, and latitudinal gradient (15° to 32°N and straddling Nearctic and Neotropical realms; Rzedowski 1978; Challenger 1998; Medellín et al. 2008; Ceballos et al. 2014). It has been reported that 7 species hibernate in belowground roosts, such as natural and human-made caves and abandoned mines (Hall and Dalquest 1963; Villa 1966; López-Wilchis 1989; Ávila-Flores 2000; López-González and Torres-Morales 2004; Ayala-Berdon and Solís-Cárdenas 2017; Aguilar-Rodríguez et al. 2021), and 1 species (*Lasiurus cinereus*) hibernates aboveground in foliage (Marín et al. 2021). In central Mexico, *Myotis velifer* moves seasonally across an altitudinal gradient from summer roosts at lower to winter roosts at higher elevations where they can hibernate from late September to early March, forming larger aggregations during December and January (Villa 1966; Camacho 2004; Ayala-Berdon and Solís-Cárdenas 2017). On the other hand, *Corynorhinus mexicanus* and *C. townsendii* typically roost singly in central and northern Mexico (Hall and Dalquest 1963; López-Wilchis 1989; López-González and Torres-Morales 2004). *Perimyotis subflavus* hibernates at high altitudes in eastern states in central and northern Mexico, and roosts singly (Hall and Dalquest 1963; Vargas 1998)—while *M. volans*, *M. californicus*, and *M. thysanodes* hibernate in central Mexico (Ávila-Flores 2000; Aguilar-Rodríguez et al. 2021).

Although other vespertilionid species in Mexico also hibernate in the United States and Canada (Perkins et al. 1990; Boyles et al. 2006; Brack 2007; Hendricks 2012; Haase et al. 2020), information on winter behavior and microclimates use by hibernating bats in temperate Mexico is limited (Ávila-Flores 2000; Ayala-Berdon and Solís-Cárdenas 2017; Marín et al. 2021). Likewise, data on hibernaculum locations are missing. In contrast to western United States where more than 1,200 hibernacula are known (Weller et al. 2018), Mexico has only 24 records (i.e., Villa 1966; López-González and Torres-Morales 2004). Therefore, clarifying which species hibernate and how abundant they are, where they hibernate, and which microclimates they use is crucial for developing conservation and management strategies.

Populations of some species of cave-hibernating bats in North America have declined dramatically since the advent of the white-nose syndrome disease (hereafter WNS), caused by the fungal pathogen *Pseudogymnoascus destructans* (hereafter Pd; Blehert et al. 2009; Cheng et al. 2021; WNSRT 2022). The fungus has spread throughout much of the United States, and in April 2022, it was found in caves in Texas, on the border with Mexico (WNSRT 2022). In addition, bats with WNS have been reported in several Texas counties less than 100 km from Mexico (WNSRT 2022). A recent study on the survival and viability of Pd conidia suggested that it can persist under elevated temperatures, facilitating long-distance dispersal in warmer conditions (Campbell et al. 2020). Furthermore, while suitable climatic conditions for WNS have been modeled in Mexico (Sierra-Durán 2020; Gómez-Rodríguez et al. 2022) there currently is no evidence that the fungus or the disease are present in Mexico. However, considering the proximity of infected hibernacula in the United States, the entry of Pd in Mexico is imminent, while potential impacts of the disease on bats remain uncertain. Shorter cold winter conditions with milder temperatures, possible availability of insects, and shorter periods of torpor of bats at subtropical latitudes might attenuate the impact of WNS (Bernard and McCracken 2017; Holz et al. 2019; Meierhofer et al. 2019a) on Mexican bat populations. Given that Mexico holds the southernmost hibernating bats in North America, a better understanding of the use of hibernation by Mexican bat populations must be considered in WNS response efforts.

For this work, we studied hibernating bats in northern and central Mexico, obtaining data on: species richness and abundance; adjacent substrate temperature of torpid bats; and the relationship between hibernation and fur temperature. Our aims were to document and describe features of hibernacula including winter behavior (such as abundance and roost pattern), and microclimates (estimated as adjacent substrate temperature) of cave-hibernating vespertilionid bats in temperate Mexico.

## Materials and methods

### Study area

We visited previously reported hibernacula and offered small rewards to colleagues, landowners, and people from rural communities for information that directed us to any new hibernacula. We explored 155 caves in 21 sites at high altitudes (>1,000 m a.s.l.) in the Sierra Madre Occidental (SMOc), the Sierra Madre Oriental (SMOr), and the Trans-Mexican Volcanic Belt (TMVB) mountain ranges. These sites were located in 11 states in central and northern Mexico, and each site includes 1 or more caves that are not more than 10 km apart. The SMOc runs north to south, 31° to 21°N, parallel to the Pacific coast, with 37.4% of its territory above 2,000 m a.s.l. and is the southern extension of the Rocky Mountains in the United States (INEGI 2001; Ferrrusquía-Villafranca et al. 2005; González-Elizondo et al. 2012). The SMOr runs north to south, 30° to 20°N, parallel to the Gulf of Mexico coast, with 10.5% of its territory above 2,000 m a.s.l. (Challenger 1998; INEGI 2001; Ferrrusquía-Villafranca et al. 2005). In central Mexico, where the SMOc and SMOr end, the TMVB crosses east-west (coast-to-coast) as far south as 18°N. The TMVB has the highest mountain peaks in Mexico (>5,000 m a.s.l.), with 44.4% of its territory over 2,000 m a.s.l. (Challenger 1998; INEGI 2001). It is important to note that we only explored sites in the northern part of the SMOc and that although we comparatively explored more sites along the SMOr and TMVB, there are still many mountainous areas to cover. The visited caves were in conifer forest (*n* = 38), oak forest (*n* = 48), xerophytic scrub (*n* = 61), and secondary vegetation modified from conifer forest (*n* = 8).

Our fieldwork included 5 consecutive periods in the coldest months during autumn and winter, hereafter considered as winter: (i) January 2018; (ii) January to March 2019; (iii) September 2019 to March 2020; (iv) September 2020 to March 2021; and (v) October 2021 to March 2022. Each winter we added between 1 and 7 sites to our evaluation. We visited each site once during the first 2 winters. In subsequent years some sites in the TMVB were visited 2 to 5 times each winter. On particular occasions we visited some sites for 2 consecutive days.

### Data collection

We recorded the cave coordinates and altitude using a GPS (Garmin Etrex 30x), and the surrounding vegetation type according to Rzedowski’s (1978) classification, which was conifer forest, oak forest, xerophytic scrub, or secondary vegetation. We also measured the length of caves using a laser distance device (Bosch GLL30).

We recorded species and abundance of torpid bats at each visit visually and with digital photographs (Meretsky et al. 2010; Loeb et al. 2015). We determined roost pattern of bats as solitarily or in cluster (≥2 bats in direct contact). Some individuals were handled and identified as species using the field guides of Medellín et al. (2008) and Morgan et al. (2019). When species identification was impossible because bats were unreachable or in order to avoid handling during the pandemic caused by SARS-CoV-2, we identified individuals to genus level. We identified bats as torpid when they were motionless and cold, with ears rolled into a rams-horns position, and/or with condensed water droplets on fur. To document specific microclimates used by torpid bats, we measured adjacent substrate temperature (*T*_sub_) and bat fur surface temperature (*T*_fur_) using 2 infrared thermometers (Extech Instruments, Models IR400 and 42545, Nashua, New Hampshire; distance to spot size 50:1 and 8:1, detection range −20 °C to 332 °C, accuracy ± [2% of reading + 2 °C], resolution of 0.1 °C, operating temperature 0 °C to 50 °C) to minimize time and disturbance inside the hibernacula. We took temperature measurements between 7:00 and 18:00 h. *T*_sub_ was taken between 1 and 3 cm from the bat, and *T*_fur_ was taken from the back of each bat, both measurements at a maximum distance of 20 cm to ensure a diameter < 2.5 cm detection area for the thermometers. When bats were in clusters, we measured temperatures of 2 to 5 individuals located in the periphery per each cluster (the largest cluster surveyed was 200 bats).

We followed the USGS National Wildlife Health Center decontamination protocol for WNS (NWHC 2016) and the Laboratorio de Ecología y Conservación de Vertebrados Terrestres field protocol for minimizing the spread of Pd fungus and avoid disturbing hibernating bats. We used gloves and decontaminated both clothing and equipment with ethanol (70%) before and after surveying a roost. We also changed clothes between roosts located in different sites and carried out our fieldwork in central Mexico toward sites progressively closer to those with Pd confirmed, traveling from southern latitudes to northern latitudes. During the last 2 winters (between September 2020 and March 2022), we also followed recommendations of the International Union for Conservation of Nature Bat Specialist Group (Kingston et al. 2021) to reduce the risk of already low probabilities of transmission of SARS-CoV-2 from humans to bats. Fieldwork was done under a scientific collecting permit issued by the Dirección General de Vida Silvestre number SGPA/DGVS/08072/21 and was performed following Sikes et al. (2016). To avoid disturbing torpid bats, we reduced team size, working time, noise and light working levels, and bat handling during surveys (Loeb et al. 2015). Throughout our study we have collaborated with 25 organizations including ejidos, agricultural communities, universities, private and ecotourism companies, civil associations, and state and federal agencies in Mexico and the United States.

### Data analyses

As a part of the documentation of hibernacula, a Chi-square test was calculated to determine whether the frequencies of hibernacula and non-hibernacula differed among vegetation types. Given that most residuals of our data on *T*_sub_ and *T*_fur_ did not show normal distribution or variance homogeneity, we performed nonparametric tests using the median as the central tendency measure. We calculated median values of *T*_sub_ and *T*_fur_ of each bat species based on the measurement of all individuals of that species (approximation per individual). Likewise, we used a Spearman rank correlation to determine how *T*_fur_ varied with *T*_sub_ based on temperature data of all bats recorded. To compare *T*_sub_ used by different species, we used data per species per hibernaculum during each visit (approximation per survey) to avoid pseudoreplication (Meierhofer et al. 2019a). The comparison was performed for those species with at least 25 surveys, using the Kruskal–Wallis test followed by a Dunn test with a Bonferroni adjustment of α/3. The level of statistical significance considered was below α = 0.05. For all analyses we used R 4.0.2 (R Core Team 2021).

## Results

**Fig. 1.**
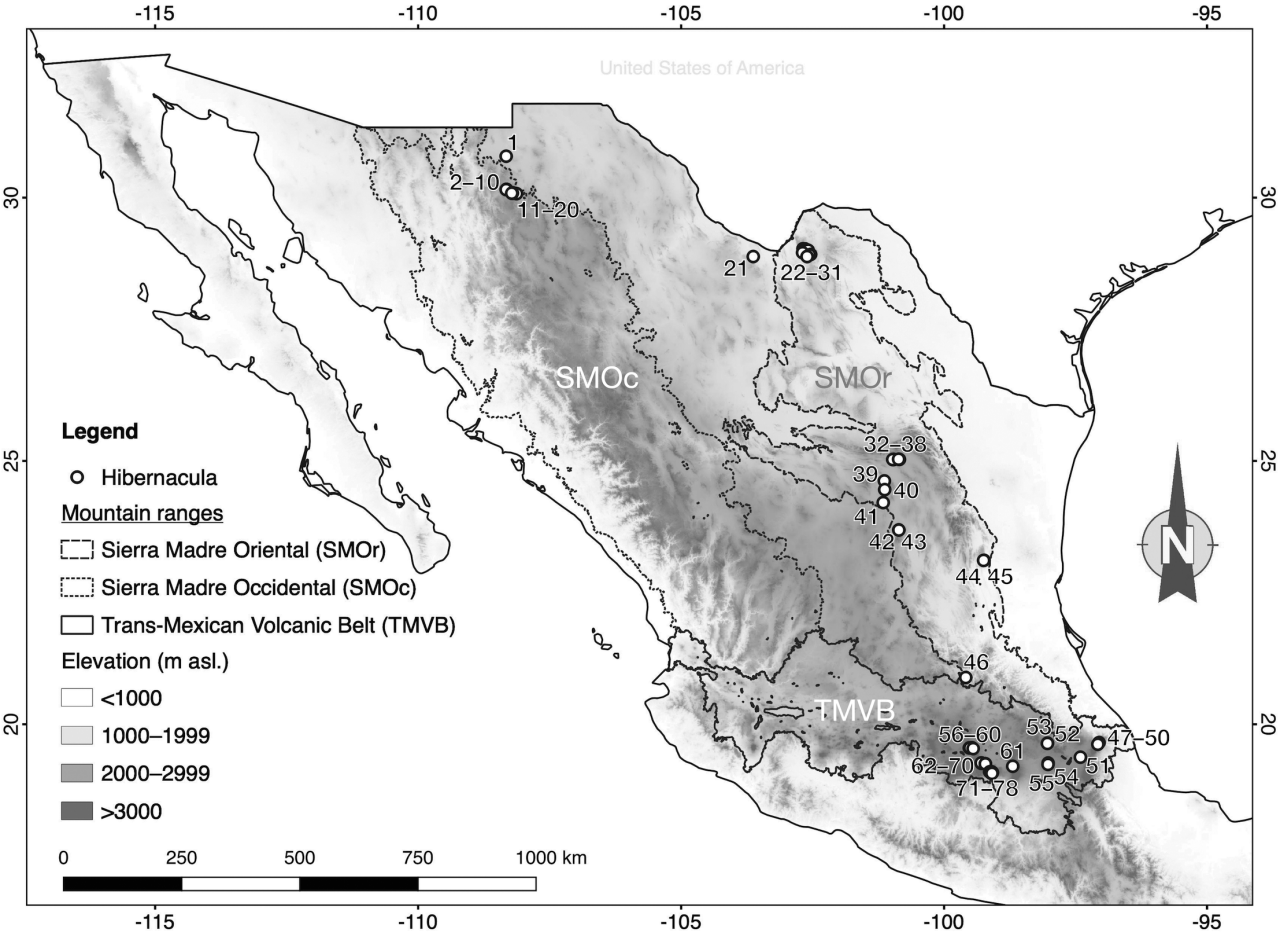
Hibernacula located in 3 mountain ranges along northern and central Mexico. Hibernaculum identification numbers correspond to those listed in Table 1.

We visited 155 caves and found 78 belowground hibernacula with torpid vespertilionid bats, while 77 caves without bats were considered non-hibernacula. The hibernacula were in central and northern Mexico, from 19.06° to 30.78°N latitude (Table 1; Fig. 1), and only 8 of these were previously reported (Hall and Dalquest 1963; Villa 1966; López-Wilchis 1989; Vargas 1998; Ávila-Flores 2000; Ayala-Berdon and Solís-Cárdenas 2017; Aguilar-Rodríguez et al. 2021). Thirty-two hibernacula were in TMVB at altitudes between 2,196 and 3,633 m a.s.l., from which 27 were above 2,900 m a.s.l.; 25 were in SMOr between 1,137 and 2,725 m a.s.l.; and 21 were in SMOc between 1,049 and 2,472 m a.s.l. (Table 1; Fig. 1). Hibernacula were located in conifer forest (*n* = 26), oak forest (*n* = 29), xerophytic scrub (*n* = 15), and secondary vegetation modified from conifer forest (*n* = 8; Table 2). There was a significant difference in the frequency of hibernacula versus non-hibernacula among vegetation types (χ^2^ = 31, *P* < 0.001), where higher ratios of hibernacula were found in conifer forest (26 of 38 caves; 68.4%), oak forest (29 of 48 caves; 60.4%), and secondary vegetation (8 of 8 caves; 100%), with fewer in xerophytic scrub (15 of 61 caves; 24.6%). Forty-eight hibernacula had lengths <50 m, 26 were 54 to 280 m, and 4 had lengths >650 m; the longest cave was 1,520 m. Half of the hibernacula were occupied by only 1 species, mainly genus *Corynorhinus* (*n* = 33), 23 had 2 species, 14 had 3 species, and 2 had 4 species with lengths of 77 and 184 m. Four hibernacula had over 200 individuals and their lengths were 17, 112, 150, and 1,339 m. Fifty-one hibernacula were natural caves and tunnels, and 27 were human-made structures such as abandoned mines and culverts. Thirty-eight hibernacula are under federal or state protection.

**Table 1.** Hibernacula recorded in Mexico during the current study. Biogeographic region: SMOc, Sierra Madre Occidental; SMOr, Sierra Madre Oriental; TMVB, Trans-Mexican Volcanic Belt. Origin: Nat, natural; Hum, Human-made. Vegetation: XS, xerophytic scrub; OF, oak forest; CF, conifer forest; SV, secondary vegetation from pine forest. Bat species: COSP, *Corynorhinus* sp.; COME, *C. mexicanus*; COTO, *C. townsendii*; EPFU, *Eptesicus fuscus*; MYSP, *Myotis* sp.; MYCI, *M. ciliolabrum*; MYOC, *M. occultus*; MYTH, *M. thysanodes*; MYVE, *M. velifer*; MYVO, *M. volans*; MYYU, *M. yumanensis*; PESU, *Perimyotis subflavus*. Superscript numbers indicate hibernacula previously reported by literature and visited for this study. For each hibernaculum we include the maximum abundance of individuals per bat species recorded between 1 and 10 visits along this study.

Hibernaculum name		Biogeographic region	Origin	Length (m)	Latitude North	Altitude (m a.s.l.)	Vegetation	COTO	COME	EPFU	MYVE	Other species
1	Tres Álamos	SMOc	Nat	200	30.8	1,421	XS	1	—	—	—	—
2	Ventanas	SMOc	Nat	23	30.1	1,864	OF	—	—	—	—	COSP (1)
3	Zorrillo	SMOc	Nat	60	30.1	1,890	OF	1	—	—	—	MYYU (1)
4	Zorrillito I	SMOc	Nat	48	30.1	1,890	OF	2	1	—	—	—
5	Zorrillito II	SMOc	Nat	32	30.1	1,871	OF	—	2	—	—	COSP (3)
6	Golondrinas Norte	SMOc	Nat	21	30.1	1,817	OF	1	—	—	—	—
7	Golondrinas Sur	SMOc	Nat	25	30.1	1,892	OF	1	—	—	—	—
8	Tadarida	SMOc	Nat	25	30.1	1,816	OF	—	—	—	1	—
9	Rincón II	SMOc	Nat	10	30.1	1,890	OF	—	—	—	—	COSP (1)
10	Salitre	SMOc	Nat	30	30.1	1,884	OF	4	4	—	—	—
11	Juana	SMOc	Nat	15	30.1	2,044	OF	—	—	—	—	COSP (1)
12	Strober A	SMOc	Nat	26	30.1	2,018	OF	—	—	—	—	COSP (6)
13	Strober A2	SMOc	Nat	16	30.1	2,072	OF	—	—	—	—	COSP (1)
14	Strober B2	SMOc	Nat	25	30.1	2,024	OF	—	—	—	—	MYVO (1), COSP (5)
15	Strober C	SMOc	Nat	17	30.1	2,028	OF	—	—	—	—	COSP (1)
16	Strober E	SMOc	Nat	30	30.1	2,064	OF	—	—	—	—	COSP (1), MYSP (1)
17	Strober F	SMOc	Nat	25	30.1	2,070	OF	—	—	—	—	COSP (1)
18	Strober G	SMOc	Nat	24	30.1	2,068	OF	—	—	—	—	COSP (1), MYSP (1)
19	Strober T	SMOc	Nat	36	30.1	2,034	OF	3	—	—	—	COSP (6), MYSP (3)
20	Arroyo	SMOc	Nat	5	30.1	2,003	OF	—	—	—	—	COSP (1)
21	Murciélago-SE	SMOc	Nat	28	28.9	1,110	XS	2	—	—	—	—
22	Caballo	SMOr	Hum	35	29.0	1,319	XS	1	—	—	—	—
23	Sal I	SMOr	Nat	41	29.0	1,971	OF	1	—	—	—	MYTH (3)
24	Sal II	SMOr	Nat	30	29.0	1,971	OF	5	—	—	—	MYTH (3)
25	Media Luna	SMOr	Nat	20	29.0	1,421	OF	3	—	—	—	—
26	Escondida-MdC	SMOr	Nat	65	29.0	2,477	CF	33	—	4	—	MYTH (2)
27	Tanquecitos	SMOr	Nat	4	28.9	1,174	XS	1	—	—	—	—
28	Juárez	SMOr	Hum	85	28.9	1,638	OF	1	—	—	—	—
29	Chamacueros I	SMOr	Hum	16	28.9	1,743	XS	4	—	—	—	—
30	Enlajados I	SMOr	Hum	15	28.8	1,512	XS	2	—	—	—	—
31	Enlajados II	SMOr	Hum	14	28.8	1,514	XS	2	—	—	—	—
32	Lechuzas	SMOr	Hum	72	25.2	2,254	CF	1	—	—	—	—
33	San Matías I	SMOr	Hum	102	25.2	2,413	CF	14	—	—	—	MYTH (3)
34	San Matías II	SMOr	Hum	33	25.2	2,413	CF	1	—	—	—	—
35	Inclinado I	SMOr	Hum	40	25.2	2,401	XS	5	—	—	—	—
36	Inclinado II	SMOr	Hum	55	25.2	2,401	XS	2	—	—	—	—
37	Chapultepec	SMOr	Hum	27	25.2	2,219	SV	1	—	—	—	—
38	Guano	SMOr	Nat	58	25.2	2,638	XS	6	—	—	—	MYCI (1)
39	Chiquihuite	SMOr	Nat	150	24.6	2,733	CF	8	—	1	—	MYTH (17)
40	Pino	SMOr	Hum	48	24.4	2,550	XS	3	—	—	—	—
41	Todos Santos	SMOr	Hum	120	24.2	2,634	XS	6	—	—	—	MYSP (1)
42	Refugio	SMOr	Hum	1000	23.6	2,634	XS	13	—	7	—	MYCI (1)
43	Santa Ana	SMOr	Hum	1520	23.6	2,725	XS	13	—	4	—	MYCI (9)
44	Chorrito	SMOr	Nat	32	23.1	1,947	CF	—	7	—	—	—
45	Charco de la Perra^1^	SMOr	Nat	115	23.1	2,091	CF	—	2	—	—	PESU (7)
46	Socorro	SMOr	Hum	6	20.8	2,310	CF	2	—	—	—	—
47	Torre de Luz	TMVB	Nat	127	19.6	2,196	SV	—	—	—	—	MYSP (1)
48	Piñas	TMVB	Nat	150	19.6	2,253	CF	—	4	—	700	PESU (8), MYSP (25)
49	Escalera	TMVB	Nat	1339	19.6	2,462	CF	—	38	—	403	PESU (6), MYSP (1)
50	Volcancillo^2^	TMVB	Nat	685	19.6	2,575	CF	—	3	—	113	PESU (3), MYSP (11)
51	Preciosa	TMVB	Hum	200	19.3	2,414	XS	—	—	—	1	MYVO (1), COSP (3), MYSP (18)
52	Túnel-Pue^3^	TMVB	Hum	270	19.6	3,163	CF	—	7	—	32	—
53	Túnel-Tlax^3^	TMVB	Hum	300	19.6	3,187	CF	—	18	—	28	—
54	Bañito^4^	TMVB	Nat	8	19.2	3,370	CF	—	5	—	52	—
55	Principal^5^	TMVB	Hum	23	19.2	3,658	CF	—	2	—	68	MYVO (1)
56	Caidení I^6^	TMVB	Hum	41	19.5	3,379	CF	2	10	—	68	—
57	Caidení II^6^	TMVB	Hum	44	19.5	3,360	CF	1	2	—	111	MYSP (4)
58	Cerro Catedral	TMVB	Nat	5	19.5	3,651	CF	—	—	—	—	COSP (1)
59	Barranca Honda	TMVB	Hum	69	19.5	3,495	CF	—	4	—	139	COSP (8)
60	Sehuayan	TMVB	Hum	15	19.5	3,210	CF	—	1	—	1	—
61	Trancas	TMVB	Hum	54	19.1	3,517	CF	—	—	—	2	—
62	Tarumba	TMVB	Hum	184	19.2	3,404	CF	1	1	—	65	MYOC (24)
63	Sepulturas	TMVB	Hum	116	19.2	3,429	CF	—	6	—	8	MYOC (4), COSP (2)
64	TN99	TMVB	Nat	10	19.2	3,240	CF	—	1	—	—	—
65	Murciélago-Xi	TMVB	Nat	270	19.2	2,916	OF	—	—	—	6	COSP (5)
66	Wendy	TMVB	Nat	17	19.2	2,924	OF	—	—	—	—	COSP (2)
67	Virgen	TMVB	Nat	240	19.2	2,931	OF	—	2	—	6	—
68	Tepozán	TMVB	Nat	280	19.2	2,942	OF	—	—	1	41	—
69	Tecuexcomac	TMVB	Nat	112	19.2	2,950	OF	—	—	—	231	—
70	Camarote	TMVB	Nat	48	19.2	2,940	OF	—	—	—	—	COSP (1)
71	Sapo	TMVB	Nat	77	19.0	3,320	CF	1	3	1	11	—
72	Escondida-MA	TMVB	Nat	17	19.0	3,321	CF	—	—	2	349	—
73	Sillón	TMVB	Nat	11	19.0	3,024	SV	—	—	—	2	—
74	Salón México	TMVB	Nat	98	19.0	3,009	SV	9	2	—	8	—
75	Troneras I	TMVB	Nat	34	19.0	3,033	SV	—	—	—	3	—
76	Troneras II	TMVB	Nat	7	19.0	3,028	SV	1	—	—	—	—
77	Sariachi	TMVB	Nat	48	19.0	3,020	SV	2	1	—	30	—
78	Ulises	TMVB	Nat	17	19.0	3,013	SV	1	—	—	1	COSP (1)

^1^ Vargas-Contreras (1998);

^2^ Hall and Dalquest (1963);

^3^ López-Wilchis (1989);

^4^ Aguilar-Rodríguez et al. (2021);

^5^ Ayala-Berdón and Solís-Cárdenas (2017);

^6^ Ávila-Flores (2000).

**Table 2.** Number of hibernacula used by the bat species recorded by biogeographic region and vegetation type. Biogeographic region: SMOc, Sierra Madre Occidental; SMOr, Sierra Madre Oriental; TMVB, Trans-Mexican Volcanic Belt. Vegetation: XS, xerophytic scrub; OF, oak forest; CF, conifer forest; SV, secondary vegetation from pine forest. Bat species: COTO, *Corynorhinus townsendii*; COME, *C. mexicanus*; EPFU, *Eptesicus fuscus*; MYCI, *Myotis ciliolabrum*; MYOC, *M. occultus*; MYTH, *M. thysanodes*; MYVE, *M. velifer*; MYVO, *M. volans*; MYYU, *M. yumanensis*; PESU, *Perimyotis subflavus*. The highest numbers of hibernacula by bat species according to each feature are in bold. We include the total number of hibernacula by bat species and cave features with their respective percentages in parentheses.

Features	Bat species										Total hibernacula (%)
	COTO	COME	EPFU	MYCI	MYTH	MYOC	MYVE	MYVO	MYYU	PESU	
Biogeographic region											
SMOc	8	3					1	1	1		21 (26.9)
SMOr	**23**	2	4	3	5					1	25 (32.1)
TMVB	8	**18**	3			2	**26**	2		3	32 (41)
Vegetation type											
CF	10	**17**	4		3	2	**16**	1		4	26 (33.3)
OF	10	4	1		2		5	1	1		29 (37.2)
XS	**14**		2	3			1	1			15 (19.2)
SV	5	2					5				8 (10.3)
Total hibernacula (%)	39 (50)	23 (29.5)	7 (9)	3 (3.8)	5 (6.4)	2 (2.6)	27 (34.6)	3 (3.8)	1 (1.3)	4 (5.1)	78 (100)

We recorded 6,089 torpid bats of at least 10 vespertilionid species (439 individuals were identified only to genus level). Of the 5,650 bats identified to species (92.8% of total torpid bats), *M. velifer* was the most abundant species in our study with 5,167 individuals. *Myotis velifer* were distributed in 27 hibernacula, from which 16 hibernacula were in conifer forests and 26 in TMVB (Table 2). Considering bat surveys when *M. velifer* was found, its maximum numbers per hibernaculum were typically of tens to a few thousand bats (range = 1 to 700 individuals, median = 9). Torpid bats were observed between late September and early March, with the largest concentrations (*n* > 100) most often found between November and January (Table 3). Regarding its roost pattern, *M. velifer* was found hibernating both solitarily and in clusters of up to 200 individuals. One hundred fifty-five *C. mexicanus* were observed in 23 hibernacula, mostly in conifer forest (*n* = 17) and in TMVB (*n* = 18), similar to *M. velifer* (Table 2). Notably, these 2 species were found together in 17 hibernacula in TMVB. For *C. townsendii* we found 190 individuals in 39 hibernacula, but in contrast with the previous species, hibernacula were mostly found in xerophytic scrub (*n* = 14) and in SMOr (*n* = 23; Table 2). Maximum numbers of *C. mexicanus* and *C. townsendii* typically were less than 10 bats per hibernaculum (range = 1 to 38 individuals, median = 2). Torpid *Corynorhinus* bats were recorded between early October and early March, and most were hibernating solitarily, with occasional clusters of 2 or 3 individuals and in 1 case 14 bats. In addition, we observed 11 individuals of *M. ciliolabrum*, 28 *M. thysanodes*, 45 *M. occultus*, 3 *M. volans*, 1 *M. yumanensis*, 23 *Eptesicus fuscus*, and 27 *P. subflavus* using from 1 to 7 hibernacula (Table 2), and with maximum numbers usually less than 10 bats per hibernacula (range = 1 to 24 individuals, median = 2), mainly hibernating solitarily.

**Table 3.** Abundance of *Myotis velifer* (left of slash) and *Corynorhinus* spp. (right of slash) hibernating in 5 caves located in central Mexico (states are in parentheses), which were visited multiple times between September and March in different years. Asterisk indicates abundance of *M. velifer* and *M. occultus* individuals considered together since some bats were unreachable and species identification was impossible.

Hibernacula	Winter	September	October	November	December	January	February
Caidení I (Estado de México)	2018 to 2019						5/3
	2019 to 2020	1/0		26/0	68/2	2/2	6/10
	2020 to 2021	0/0			5/7		
	2021 to 2022		0/2		16/0		
Caidení II (Estado de México)	2018 to 2019						18/1
	2019 to 2020	3/0		111/2	87/1	59/0	29/2
	2020 to 2021	4/0			29/1		
	2021 to 2022		9/1		81/1		
Tarumba* (Ciudad de México)	2017 to 2018					87/1	
	2018 to 2019					59/0	
	2019 to 2020	11/0		39/1		56/2	
	2020 to 2021	3/1			41/0		
	2021 to 2022		10/0		48/0		72/1
Escondida (Ciudad de México)	2018 to 2019					277/0	
	2019 to 2020	34/0		349/0		279/0	
	2020 to 2021	27/0		237/0	167/0		
	2021 to 2022		69/0		185/0		
Sapo (Ciudad de México)	2018 to 2019					10/3	
	2019 to 2020	0/0		7/0		2/1	
	2020 to 2021	3/0		11/1	6/1		
	2021 to 2022		3/0		5/2		

**Fig. 2.**
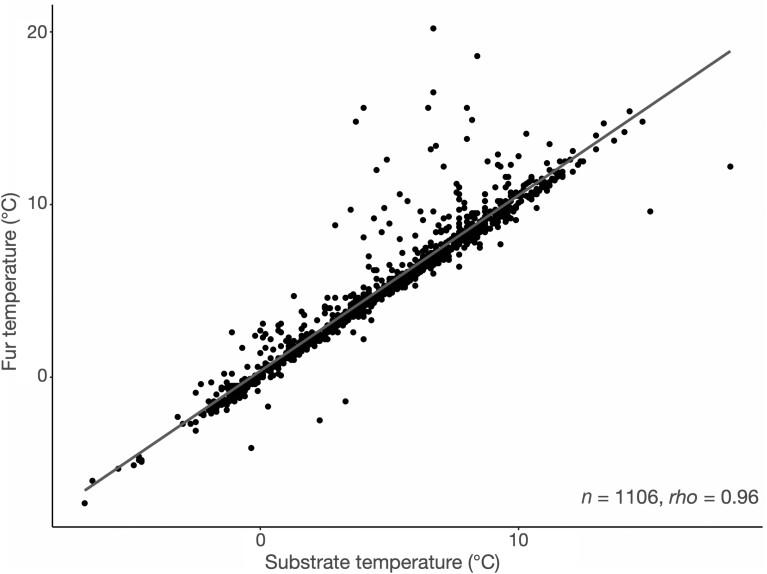
Relationship between fur and adjacent substrate temperatures for all cave-hibernating bats found in northern and central Mexico.

**Fig. 3.**
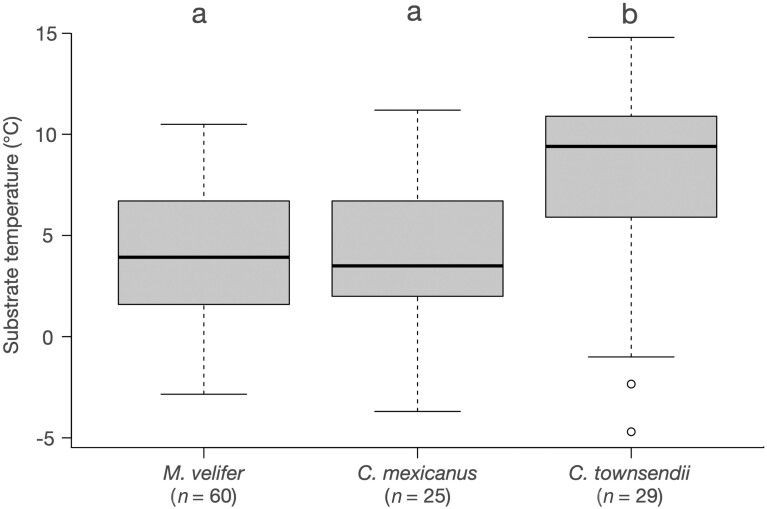
Adjacent substrate temperatures (in °C) in 3 species of hibernating bats (number of surveys in parentheses). Different letters indicate significant differences among species (*P* < 0.025; Dunn test considering a Bonferroni adjustment of α/3).

We recorded *T*_sub_ and *T*_fur_ of 1,106 vespertilionid bats that were identified to species (18.6% of total torpid bats) during 90 surveys across 4 winters between January 2019 and March 2022 (Table 4). We obtained *T*_sub_ and *T*_fur_ medians from 9 species of 4 genera. The median of the absolute difference between *T*_fur_ and *T*_sub_ was 0.2 °C (Q1 = 0.1, Q3 = 0.5). Only the 3 most abundant species (*M. velifer*, *C. mexicanus*, and *C. townsendii*) had enough surveys to be compared. Temperature measures were recorded for 863 *M. velifer* in 24 hibernacula visited from 1 to 7 times (60 surveys), 89 *C. mexicanus* in 17 hibernacula visited from 1 to 5 times (25 surveys), and 80 *C. townsendii* in 25 hibernacula visited 1 or 2 times (29 surveys). The other 6 bat species were found ≤5 times each and temperature data were obtained from 2 to 26 individuals (Table 4). We found a strong and positive correlation between *T*_sub_ and *T*_fur_ (*rho* = 0.96, *P* < 0.001; Fig. 2). All bats were hibernating at *T*_sub_ between −6.8 °C and 18.6 °C, and at *T*_fur_ between −7.3 °C and 20.2 °C, where more than 95% of measured individuals had *T*_fur_ < 12 °C. *Corynorhinus townsendii* was found using higher *T*_sub_ than *M. velifer* (*D* = 4.41, df = 2, *P* < 0.001) and *C. mexicanus* (*D* = −3.6, *P* < 0.001; Fig. 3).

**Table 4.** Adjacent substrate (*T*_sub_) and fur (*T*_fur_) temperatures (in °C) of 9 species of torpid bats reported in this study in central and northern Mexico.

Bat species	*n*	*T*_sub_ (°C)					*T*_fur_ (°C)				
		Min	Q1	Median	Q3	Max	Min	Q1	Median	Q3	Max
*Corynorhinus mexicanus*	89	−4.9	3.5	7.1	8.7	11.2	−5.1	4.6	8.2	9.6	14.9
*Corynorhinus townsendii*	80	−5.5	6.8	9.2	10.8	14.8	−5.3	7.5	9.6	11.2	15.4
*Eptesicus fuscus*	10	−0.2	3.2	6.7	8.5	10.9	0.1	2.5	6.8	8.5	11
*Myotis ciliolabrum*	9	5.6	6.8	7.3	8.2	12.3	5.6	7.2	7.4	8.3	12.3
*Myotis occultus*	26	0	1.6	2.9	4	5.5	0.1	1.9	3	4.2	5.6
*Myotis thysanodes*	14	7.1	7.3	8.35	10.3	11.4	7.1	7.8	8.8	10.7	14.1
*Myotis velifer*	863	−6.8	1.2	3.9	6.8	18.2	−7.3	1.5	4.2	7.2	20.2
*Myotis volans*	2	8.5	NA	8.6	NA	8.7	8.7	NA	8.7	NA	8.7
*Perimyotis subflavus*	13	5.5	7.3	8.4	9.6	10.3	6.2	7.8	8.7	10	10.5

## Discussion

Although most of our visits to hibernacula covered only 1 day of evaluation, we considered that observed torpid bats during our study were likely hibernating, based on multiple lines of evidence. Considering that skin temperature may be a proxy of body temperature, and that skin and fur temperatures are relatively similar at low body temperatures (Bartonička et al. 2017; Geiser 2021), the *T*_fur_ of torpid bats indicated hibernation rather than daily torpor given that they were very low (*T*_fur_ < 12 °C). On some occasions we visited a cave for 2 consecutive days and we were able to record torpid bats of *M. velifer*, *C. mexicanus*, and *P. subflavus* in the same place inside the cave (similar to observations of Hall and Dalquest 1963). Camera traps placed in front of torpid *M. velifer* in hibernacula in central Mexico recorded them as inactive during several consecutive days between late September and early March (Ramos-H. et al. 2022). Finally, all torpid bat species we found have been previously reported hibernating in Mexico (Hall and Dalquest 1963; Villa 1966; López-González and Torres-Morales 2004; Aguilar-Rodríguez et al. 2021) and in the United States (Perkins et al. 1990; Brack 2007; Hendricks 2012; Meierhofer et al. 2019a; Haase et al. 2020). For *M. occultus* we are considering reports on its sister species *M. lucifugus* (Piaggio et al. 2002).

Therefore, here we provide relevant information on geographic locations of hibernacula, winter behavior, and specific microclimates for 10 temperate vespertilionid bat species in the southernmost occurrence of bat hibernation in North America. We report the first hibernation record in Mexico for *E. fuscus*, *M. ciliolabrum*, *M. occultus*, and *M. yumanensis*. We also add 70 new hibernacula to those 24 previously known for the country. Thus, in 5 consecutive evaluated winters we have increased by over 50% the number of cave-hibernating bat species and have quadrupled the number of hibernacula for Mexico.

In our study, hibernacula and large numbers of torpid bats were more commonly found in caves in oak and conifer forests. These forests are usually located at higher altitudes in temperate mountain areas of Mexico, where comparatively higher humidity and lower environmental temperature are found (Rzedowski 1978; Ferrusquía-Villafranca et al. 2005). Likewise, it has been reported that bats in temperate regions, including *M. velifer* in central Mexico (Villa 1966; Camacho 2004), select caves at higher altitudes to hibernate because of their optimal microclimates (McGuire and Boyle 2013). Both vegetation type and internal cave temperature are strongly linked with external environmental temperature (Geiger et al. 1995; Perry 2013; Meierhofer et al. 2019a). Therefore, our observations suggest that conifer and oak forests in temperate Mexico would occur in the same altitudinal levels as caves used by bats to hibernate. It is important to note that we found more hibernacula located at higher altitudes in central Mexico compared to northern latitudes, which may be associated with decrease in environmental temperature with latitude (Montgomery 2006). Thus, vegetation types as well as thermal cave conditions may occur at different altitudes according to latitude. It has been reported that forest cover generates more stable temperatures and humidity at ground than those in open areas located at the same altitudinal level (Geiger et al. 1995); thus, vegetation may influence cave temperature (Perry 2013; Piksa et al. 2013). However, more studies about the association of vegetation with hibernacula occurrence along latitudinal gradients are necessary.

Large caves usually hold a higher richness and abundance of bats because they may provide a wide range of microclimates (Arita 1993; Briggler and Prather 2003; Altringham 2011; Perry 2013). However, we have not found this trend in our hibernation caves; higher numbers of species or individuals were in caves with different lengths. Although this may highlight the importance of caves, regardless of their length for hibernating Mexican bats, other studies that include additional large caves are needed. On the other hand, a third of the hibernacula we report are human-made caves (abandoned mines and culverts), reflecting the capacity of bats to take advantage of human-generated structures.

Although *M. velifer* has a wide distribution in Mexico (Castro-Campillo et al. 2014) and was reported hibernating in the United States (Humphrey and Oli 2015; Caire et al. 2018; Meierhofer et al. 2019a; Haase et al. 2020), in northern Mexico we only found 1 torpid individual in a single cave. With this exception, all hibernacula of *M. velifer* were in the TMVB, mainly located in conifer forests and with numbers of tens to hundreds of bats per cave. This species previously has been reported hibernating only in central Mexico (Villa 1966; López-Wilchis 1999; Ávila-Flores 2000; Ayala-Berdon and Solís-Cárdenas 2017), and regardless of its abundance in our study, its numbers were much lower than those in the southern United States, where several hibernacula hold thousands of bats (Humphrey and Oli 2015; Caire et al. 2018).

Similar to *M. velifer*, *C. mexicanus* was found mainly hibernating in caves in conifer forests across the TMVB. Moreover, we found both species frequently sharing hibernacula, as previously reported only in central Mexico (Hall and Dalquest 1963; López-Wilchis 1999; Ávila-Flores 2000; Aguilar-Rodríguez et al. 2021), and hibernating at very similar *T*_sub_. Our findings suggest that both species may use similar microclimates for hibernation in central Mexico, as reported for bats wintering together in other regions (Nagy and Postawa 2011; Piksa et al. 2013)—raising the question of why there is a lack of records of torpid *M. velifer* in northern Mexico, even when we found *C. mexicanus* hibernating in all 3 Mexican mountain ranges we explored.

*Corynorhinus townsendii* was the most ubiquitous species found in our study and had numbers typically less than 10 bats per hibernaculum. These observations are in accordance with data provided by other studies in the western United States (Hendricks 2012; Weller et al. 2018; Whiting et al. 2018), although those documented a few thousand individuals in some caves. Unlike *M. velifer* and *C. mexicanus*, *C. townsendii* seems more flexible in hibernacula use, given that we found torpid bats in several caves in all 4 vegetation types across the 3 mountain ranges. Although we occasionally found individuals of both *Corynorhinus* species sharing hibernacula—similar to previous studies (López-González and Torres-Morales 2004; Gómez-Ruiz et al. 2006)—we found *C. townsendii* more frequently in caves in xerophytic scrub vegetation, while *C. mexicanus* were in caves in conifer forest. Different habitat affinities have also been described for both species throughout the year, as *C. townsendii* commonly inhabits lower altitudes and arid environments, while *C. mexicanus* inhabits higher altitudes with cooler and more humid environments (Handley 1959). Likewise, we observed *C. townsendii* hibernating at higher *T*_sub_ than *C. mexicanus*, suggesting that these species have different hibernacula microclimate preferences. Related bat species with morphological similarities may show differences in the use of hibernacula and microsites (Piksa et al. 2013), which may be associated with their summer habitat use (Nagy and Postawa 2011; Meierhofer et al. 2019a).

We found *P. subflavus* in hibernacula last visited and reported 30 to 70 years ago (Davis 1959; Hall and Dalquest 1963; Vargas 1998) and we recorded new hibernacula. Our numbers of *P. subflavus* seemed to be similar to those reported in caves by Briggler and Prather (2003) and be lower than the abundance noted by Meierhofer et al. (2019b), who reported hundreds and even thousands of bats in culverts. We also added new hibernacula for *M. thysanodes* and *M. volans* to those reported previously (Ávila-Flores 2000; Aguilar-Rodríguez et al. 2021). Although there are no previous records of torpid *E. fuscus* in Mexico, its hibernation was suggested by Woloszyn and Woloszyn (1982) and Segura (2010) based on significant fat accumulation in bats collected at the beginning of the winter. Although *E. fuscus*, *M. ciliolabrum*, *M. occultus*, *M. thysanodes*, *M. volans*, and *M. yumanensis* have wide distributions in central and northern Mexico (Medellín et al. 2008; Ceballos 2014), we found relatively few torpid individuals and hibernacula for these species. Like our data, studies in the western United States reported that *E. fuscus* and the other 5 *Myotis* species hibernate in small numbers, typically less than 10 bats per cave (Perkins et al. 1990; Hendricks et al. 2012; Whiting et al. 2018).

In this study, *Corynorhinus* species were usually less abundant than *Myotis* species, which contrasts with Weller et al. (2018) and Whiting et al. (2018) who reported larger numbers of *C. townsendii* in hibernacula across the western United States. In general, the abundance of torpid bats in our hibernacula was similar in size to those reported in the western United States (Perkins et al. 1990; Hendricks 2012; Weller et al. 2018; Whiting et al. 2018), with aggregations of tens to a few hundred individuals per cave. In contrast, our numbers of bats were lower than those observed in the eastern United States (prior to the arrival of WNS), where hibernacula may often hold thousands of individuals (Brack 2007; Storm and Boyles 2011; Frick et al. 2015; Langwig et al. 2015). Geographical variation in occurrence of hibernating bat species within Mexico (e.g., lack of torpid *M. velifer* in the north) and in abundances in comparison with the United States (e.g., differences in the number of torpid bats) may be due to several factors including variation in availability of areas to overwinter, bat abundances, availability and quality of roosts, use of roosts other than caves, roosts located in inaccessible areas, detectability of bats inside the roost, genetic characteristics, and historical stressors (Perkins et al. 1990; Frick et al. 2015; Weller et al. 2018; Whiting et al. 2018).

Site selection has been suggested as the main mechanism bats use to maintain a stable temperature during hibernation (Boyles et al. 2020). In addition to food availability and winter behavior, shorter and milder winters in Mexico could favor the use of microsites instead of active thermoregulation, especially if subtropical populations of hibernating bats exhibit shorter torpor bouts than northern populations (Dunbar and Brigham 2010; Geiser and Stawski 2011; Meierhofer et al. 2019a). Across all hibernacula, we found that *T*_fur_ of bats was strongly and positively correlated with *T*_sub_ and may be indicative of site selection of bats as a measure to reduce energy expenditure (Humpries et al. 2002; Kim et al. 2013; Boyles et al. 2020). However, because we did not measure temperature at different sections of the cave, it is not possible to distinguish whether bats selected for hibernacula conditions, microsites, or both. Similar to our study, a relationship between *T*_sub_ and *T*_fur_ has been reported in hibernating bats elsewhere (Meierhofer et al. 2019a), as well as very small differences of less than 1 °C between both temperatures (Storm and Boyles 2011; Kim et al. 2013). The correlation that we found in subtropical conditions is relevant in the context of the southernmost hibernation limit for Nearctic species and gives insight into thermoregulation strategies of the bats we surveyed.

Few studies have reported microclimatic temperatures used for hibernation by bat species in Mexico, and those include data on *C. mexicanus*, *C. townsendii*, *M. velifer*, and *M. volans* (López-Wilchis 1989; Ávila-Flores 2000). Although these studies used a different methodological approach, i.e., measuring ambient temperature as close as possible to the roosting surface, microclimatic temperatures provided are consistent with our findings on median *T*_sub_, ranging between 1.6 °C and 19.7 °C (López-Wilchis 1989; Ávila-Flores 2000). In general, median *T*_sub_ for the 9 species we recorded were also consistent with averages reported in several hibernating bat species in the northern United States (Brack 2007; Ingersoll et al. 2010; Storm and Boyles 2011; Langwig et al. 2016; Hopkins et al. 2021), while our maximum *T*_sub_ were similar to averages noted in the southern United States (Meierhofer et al. 2019a; Smith et al. 2021; Supplementary Data SD1). In contrast to our findings, we did not find studies that recorded hibernating bats at *T*_sub_ below zero (Kim et al. 2013; Langwig et al. 2016; Meierhofer et al. 2019a). However, there are observations of negative body and skin temperatures (≥−2.9 °C) in hibernating mammals, including bats (Geiser 2021). This is likely an indication that measurements of *T*_sub_ tend to be a few degrees lower than body temperature when ambient temperature is very cold. Finding these consistencies in *T*_sub_ suggests that bats may use a range of microclimates suitable for hibernation throughout their wide geographic distribution in North America (Webb et al. 1996).

Our study shows that belowground hibernacula at high altitudes in northern and central Mexico are an important resource for temperate vespertilionid bats during a crucial time in their lives. Although we found 10 bat species, it is likely that more species will be found hibernating in Mexico given that our surveys were restricted to 1 to 5 days during the hibernation period per cave and that there are still several areas to explore. Furthermore, bats can use hibernacula during different life cycle periods including autumn swarming, mating, and maternity, increasing their importance for conservation. Although around half of the visited roosts are in areas with some level of federal and state protection, caves are not usually considered in conservation efforts (Medellín et al. 2017). Therefore, it is necessary to continue searches for hibernacula and evaluate their potential importance throughout the year (Arita 1993; Medellín et al. 2017), prioritizing conservation and management in roosts with high abundances and richness, maintaining crucial behaviors, and sheltering endemic and threatened species.

Baseline counts of bats at hibernacula help monitor abundance and species richness and may provide an estimate of regional population sizes (Loeb et al. 2015), and coupled with understanding of the temperatures used during hibernation are crucial for developing conservation and management strategies under current climatic conditions and providing a basis for evaluating responses to future environmental changes (Brack 2007; Meierhofer et al. 2019a) and other threats such as emerging diseases (Frick et al. 2019; Cheng et al. 2021). For example, considering the ongoing expansion of WNS in North America, continuous monitoring of hibernating bat populations would play an essential role in developing and implementing biosecurity protocols to prevent Pd spread and monitoring programs to detect the pathogen as soon as possible after its introduction (Holz et al. 2019; Cheng et al. 2021).

Future studies should focus on bat hibernation in Mexico at multiple temporal and spatial scales. More detailed information on duration of the hibernation period, duration and frequency of torpor and arousal bouts, diversity and species turnover within and between hibernacula, roost pattern, and microsite selection should be assessed. Furthermore, expanding studies across larger latitudinal and altitudinal gradients in Mexico will aid in understanding the ecology and physiology of hibernation, including the effects of climate change, in the southernmost limit of its occurrence in North America. Securing the conservation of bats in hibernacula must be a priority for local, state, and federal authorities in Mexico and elsewhere.

## Supplementary data

Supplementary data are available at *Journal of Mammalogy* online.

**Supplementary Data SD1.**—Adjacent substrate (*T*_sub_) and fur (*T*_fur_) temperatures (in °C) of torpid vespertilionid bat species reported by similar studies in the United States. Mean values and standard deviations are present.

**Supplementary Data SD2.**—Extended acknowledgements for contributors to this study.

gyae027_suppl_Supplementary_Datas_SD1

gyae027_suppl_Supplementary_Datas_SD2

## References

[CIT0001] Aguilar-Rodríguez PA , Vega-Gutiérrez VH, Miguel-Méndez RS, Hernandez-Vargas AA, Cabrera-Campos I, Ayala-Berdon J. 2021. Winter occupation of two bat hibernacula in a montane ecosystem of central Mexico. Boletín de la Red Latinoamericana y del Caribe para la Conservación de los Murciélagos 12:3–9.

[CIT0002] Altringham JD. 2011. Bats, from evolution to conservation. 2nd ed. Oxford (Oxfordshire, UK): Oxford University Press.

[CIT0003] Arita HT. 1993. Conservation biology of the cave bats of Mexico. Journal of Mammalogy 74(3):693–702. 10.2307/1382291

[CIT0004] Ávila-Flores R. 2000. Patrones de uso de cuevas en murciélagos del centro de México [bachelor’s thesis]. [Tlalnepantla (State of Mexico, Mexico)]: Universidad Nacional Autónoma de México.

[CIT0005] Ayala-Berdon J , Solís-Cárdenas V. 2017. New record and site characterization of a hibernating colony of *Myotis velifer* in a mountain ecosystem of central Mexico. Therya 8(2):171–174. 10.12933/therya-17-469

[CIT0006] Bartonička T , Bandouchova H, Berková H, Blažek J, Lučan R, Horáček I, Martínková N, Pikula J, Řehák Z, Zukal J. 2017. Deeply torpid bats can change position without elevation of body temperature. Journal of Thermal Biology 63:119–123. 10.1016/j.jtherbio.2016.12.005 28010809

[CIT0007] Bernard RF , McCracken GF. 2017. Winter behavior of bats and the progression of white-nose syndrome in the southeastern United States. Ecology and Evolution 7(5):1487–1496. 10.1002/ece3.2772 28261459 PMC5330875

[CIT0008] Blehert DS , Hicks AC, Behr M, Meteyer CU, Berlowski-Zier BM, Buckles EL, Coleman JTH, Darling SR, Gargas A, Niver R, et al. 2009. Bat white-nose syndrome: an emerging fungal pathogen? Science 323(5911):227–227. 10.1126/science.1163874 18974316

[CIT0009] Boyles JG , Dunbar MB, Whitaker JO Jr. 2006. Activity following arousal in winter in North American vespertilionid bats. Mammal Review 36(4):267–280. 10.1111/j.1365-2907.2006.00095.x

[CIT0010] Boyles JG , Johnson JS, Blomberg A, Lilley TM. 2020. Optimal hibernation theory. Mammal Review 50(1):91–100. 10.1111/mam.12181

[CIT0011] Brack V Jr . 2007. Temperatures and locations used by hibernating bats, including *Myotis sodalis* (Indiana bat), in a limestone mine: implications for conservation and management. Environmental Management 40(5):739–746. 10.1007/s00267-006-0274-y 17874161

[CIT0012] Briggler JT , Prather JW. 2003. Seasonal use and selection of caves by Eastern pipistrelle bat (*Pipistrellus subflavus*). The American Midland Naturalist 149(2):406–412. 10.1674/0003-0031(2003)149[0406:suasoc]2.0.co;2

[CIT0013] Caire W , Loucks LS, Shaw JB, Evans JW, Gillies KE, Caywood MA. 2018. Variation in the number of hibernating cave myotis (*Myotis velifer*) in western Oklahoma and northwest Texas caves prior to the arrival of white-nose syndrome. The Southwestern Naturalist 63(2):124–132. 10.1894/0038-4909-63-2-124

[CIT0014] Camacho PMA. 2004. Análisis de las capturas y recapturas de *Myotis velifer* (Chiroptera: Vespertilionidae) en tres localidades del Eje Volcánico Transversal [bachelor’s thesis]. [Mexico City (Mexico)]: Universidad Autónoma Metropolitana.

[CIT0015] Campbell LJ , Walsh DP, Blehert DS, Lorch JM. 2020. Long-term survival of *Pseudogymnoascus destructans* at elevated temperatures. Journal of Wildlife Diseases 56(2):278–287. 10.7589/2019-04-106 31622188

[CIT0016] Castro-Campillo A , Gonzalez E, Aguilera, Ramirez-Pulido J. 2014. *Myotis velifer* (J.A. Allen, 1890). In: Ceballos G, editor. Mammals of Mexico. Baltimore (MD, USA): John Hopkins University Press; p. 798–800.

[CIT0017] Ceballos G , Arroyo-Cabrales J, Medellin R, Medrano-González L, Oliva G. 2014. Diversity and conservation. In: Ceballos G, editor. Mammals of Mexico. Baltimore (MD, USA): John Hopkins University Press; p. 1–44.

[CIT0018] Challenger A. 1998. Utilización y conservación de los ecosistemas terrestres de México: pasado, presente y futuro. Mexico City (Mexico): Comisión Nacional para el Uso y Conocimiento de la Biodiversidad, Instituto de Biología-UNAM, Agrupación Sierra Madre.

[CIT0019] Cheng TL , Reichard JD, Coleman JTH, Weller TJ, Thogmartin WE, Reichert BE, Bennett AB, Broders HG, Campbell J, Etchison K, et al. 2021. The scope and severity of white-nose syndrome on hibernating bats in North America. Conservation Biology 35(5):1586–1597. 10.1111/cobi.13739 33877716 PMC8518069

[CIT0020] Davis WH. 1959. Taxonomy of the eastern pipistrel. Journal of Mammalogy 40(4):521–531. 10.2307/1376268

[CIT0021] Dunbar MB , Brigham RM. 2010. Thermoregulatory variation among populations of bats along a latitudinal gradient. Journal of Comparative Physiology B 180(6):885–893. 10.1007/s00360-010-0457-y 20213177

[CIT0022] Ferrusquía-Villafranca I , Gonzáles-Guzmán LI, Cartron JLE. 2005. Northern Mexico’s landscape, part I: the physical setting and constraints on modeling biotic evolution. In: Cartron JLE, Ceballos G, Felger RS, editors. Biodiversity, ecosystems, and conservation in northern Mexico. Oxford (UK): Oxford University Press; p. 11–38.

[CIT0023] Frick WF , Kingston T, Flanders J. 2019. A review of the major threats and challenges to global bat conservation. Annals of the New York Academy of Sciences 1469(1):5–25. 10.1111/nyas.14045 30937915

[CIT0024] Frick WF , Puechmaille SJ, Hoyt JR, Nickel BA, Langwig KE, Foster JT, Barlow KE, Bartonička T, Feller D, Haarsma AJ, et al. 2015. Disease alters macroecological patterns of North American bats. Global Ecology and Biogeography 24(7):741–749. 10.1111/geb.12290

[CIT0025] Geiger R , Aron RH, Todhunter P. 1995. The climate near the ground. Lanham (MD, USA): Harvard University Press.

[CIT0026] Geiser F. 2021. Ecological physiology of daily torpor and hibernation. New York (NY, USA): Springer.

[CIT0027] Geiser F , Stawski C. 2011. Hibernation and torpor in tropical and subtropical bats in relation to energetics, extinctions, and the evolution of endothermy. Integrative and Comparative Biology 51(3):337–348. 10.1093/icb/icr042 21700575

[CIT0028] Gómez-Rodríguez RA , Sánchez-Cordero V, Boyer D, Schondube JE, Rodríguez-Moreno A, Gutiérrez-Granados G. 2022. Risk of infection of white-nose syndrome in North American vespertilionid bats in Mexico. Ecological Informatics 72:101869. 10.1016/j.ecoinf.2022.101869

[CIT0029] Gómez-Ruiz EP , López-González C, García-Mendoza DF. 2006. *Corynorhinus mexicanus* y *C. townsendii* (Chiroptera: Vespertilionidae) en la Sierra Madre Occidental. Vertebrata Mexicana 19:5–12.

[CIT0030] González-Elizondo SM , González-Elizondo M, Tena-Flores JA, Ruacho-González L, López-Enríquez IL. 2012. Vegetación de la Sierra Madre Occidental, México: una síntesis. Acta Botánica Mexicana 100:351–403.

[CIT0031] Haase CG , Fuller NW, Dzal YA, Hranac CR, Hayman DTS, Lausen CL, Silas KA, Olson SH, Plowright RK. 2020. Body mass and hibernation microclimate may predict bat susceptibility to white-nose syndrome. Ecology and Evolution 11(1):506–515. 10.1002/ece3.7070 33437446 PMC7790633

[CIT0032] Hall ER , Dalquest WW. 1963. The mammals of Veracruz. University of Kansas Publications, Museum of Natural History 14:165–362.

[CIT0033] Handley CO. 1959. A revision of American bats of the genera *Euderma* and *Plecotus*. Proceedings of the United States National Museum 110(3417):95–246. 10.5479/si.00963801.110-3417.95

[CIT0034] Hendricks P. 2012. Winter records of bats in Montana. Northwestern Naturalist 93(2):154–162. 10.1898/nwn11-20.1

[CIT0035] Holz P , Hufschmid J, Boardman WS, Cassey P, Firestone S, Lumsden LF, Prowse TAA, Reardon T, Stevenson M. 2019. Does the fungus causing white-nose syndrome pose a significant risk to Australian bats? Wildlife Research 46(8):657–668. 10.1071/WR18194

[CIT0036] Hopkins SR , Hoyt JR, White JP, Kaarakka HM, Redell JA, DePue JE, Scullon WH, Kilpatrick AM, Langwig KE. 2021. Continued preference for suboptimal habitat reduces bat survival with white-nose syndrome. Nature Communications 12(1):1–9. 10.1038/s41467-020-20416-5 PMC7794521 33420005

[CIT0037] Humphrey MM , Thomas DW, Kramer DL. 2002. The role of energy availability in mammalian hibernation: a cost-benefit approach. Physiological and Biochemical Zoology 76(2):165–179. 10.1086/367950 12794670

[CIT0038] Humphrey SR , Oli MK. 2015. Population dynamics and site fidelity of the cave bat, *Myotis velifer*, in Oklahoma. Journal of Mammalogy 96(5):946–956. 10.1093/jmammal/gyv095

[CIT0039] Ingersoll TE , Navo KW, de Valpine P. 2010. Microclimate preferences during swarming and hibernation in the Townsend’s big-eared bat, *Corynorhinus townsendii*. Journal of Mammalogy 91(5):1242–1250. 10.1644/09-mamm-a-288.1

[CIT0040] Instituto Nacional de Estadística y Geografía [INEGI]. 2001. Conjunto de datos vectoriales Fisiográficos. Continuo Nacional, escala 1:1 000 000. Serie I (Provincias fisiográficas). Aguascalientes (Aguascalientes, Mexico): Instituto Nacional de Estadística y Geografía. [accessed 24 May 2022]. https://www.inegi.org.mx/app/biblioteca/ficha.html?upc=702825267575

[CIT0041] Kim SS , Choi YS, Yoo JC. 2013. Thermal preference and hibernation period of Hodgson’s bats (*Myotis formosus*) in the temperate zone: how does the phylogenetic origin of a species affect its hibernation strategy? Canadian Journal of Zoology 91(2):47–55. 10.1139/cjz-2012-0145

[CIT0042] Kingston T , Frick W, Kading R, Leopardi S, Medellín RA, Mendenhall IH, Racey P, Shapiro JT, Vicente-Santos A, Víquez-R L, Worledge L. 2021. IUCN SSC Bat Specialist Group (BSG) recommended strategy for researchers to reduce the risk of transmission of SARS-CoV-2 from humans to bats. Gland (Vaud, Switzerland): IUCN SSC Bat Specialist Group. [accessed 21 Sep 2021]. https://www.iucnbsg.org/uploads/6/5/0/9/6509077/amp_recommendations_for_researchers_final.pdf

[CIT0043] Langwig KE , Frick WF, Hoyt JR, Parise KL, Drees KP, Kunz TH, Foster JT, Kilpatrick AM. 2016. Drivers of variation in species impacts for a multi-host fungal disease of bats. Philosophical Transactions of the Royal Society of London, Series B: Biological Sciences 371(1709):20150456. 10.1098/rstb.2015.0456 28080982 PMC5095535

[CIT0044] Langwig KE , Hoyt JR, Parise KL, Kath J, Kirk D, Frick WF, Foster JT, Kilpatrick AM. 2015. Invasion dynamics of white-nose syndrome fungus, midwestern United States, 2012–2014. Emerging Infectious Diseases 21(6):1023–1026. 10.3201/eid2106.150123 25989230 PMC4451901

[CIT0045] Loeb SC , Rodhouse TJ, Ellison LE, Lausen CL, Reichard JD, Irvine KM, Ingersoll TE, Coleman JTH, Thogmartin WE, Sauer JR, et al. 2015. A plan for the North American bat monitoring program (NABat). Asheville (NC, USA): United States Forest Service, Southern Research Station. General Technical Report SRS-208; p. 1–100.

[CIT0046] López-González C , Torres-Morales L. 2004. Use of abandoned mines by long-eared bats, genus *Corynorhinus* (Chiroptera: Vespertilionidae) in Durango, México. Journal of Mammalogy 85(5):989–994. 10.1644/bwg-124

[CIT0047] López-Wilchis R. 1989. Biología de *Plecotus mexicanus* (Chiroptera: Vespertilionidae) en el estado de Tlaxcala, México [doctoral thesis]. [Mexico City (Mexico)]: Universidad Nacional Autónoma de México.

[CIT0048] López-Wilchis R. 1999. Murciélagos asociados a una colonia de *Corynorhinus mexicanus* G. M. Allen, 1916 (Chiroptera: Vespertilionidae). Vertebrata Mexicana 5:9–16.

[CIT0049] Marín G , Ramos-H D, Cafaggi D, Sierra-Durán C, Gallegos A, Romero-Ruíz A, Medellín RA. 2021. Challenging hibernation limits of hoary bats: the southernmost record of *Lasiurus cinereus* hibernating in North America. Mammalian Biology 101(3):287–291. 10.1007/s42991-020-00080-4

[CIT0050] McGuire LP , Boyle WA. 2013. Altitudinal migration in bats: evidence, patterns, and drivers. Biological Reviews of the Cambridge Philosophical Society 88(4):767–786. 10.1111/brv.12024 23480862

[CIT0051] Medellín RA , Arita H, Sánchez O. 2008. Identificación de los murciélagos de México. 2nd ed. Mexico City (Mexico): Instituto de Ecología UNAM.

[CIT0052] Medellín RA , Wiederholt R, Lopez-Hoffman L. 2017. Conservation relevance of bat caves for biodiversity and ecosystem services. Biological Conservation 211:45–50. 10.1016/j.biocon.2017.01.012

[CIT0053] Meierhofer MB , Johnson JS, Leivers SJ, Pierce BL, Evans JE, Morrison ML. 2019a. Winter habitats of bats in Texas. PLoS One 14(8):e0220839. 10.1371/journal.pone.0220839 31393965 PMC6687166

[CIT0054] Meierhofer MB , Leivers SJ, Fern RR, Wolf LK, Young JH Jr, Pierce BL, Evans JW, Morrison ML. 2019b. Structural and environmental predictors of presence and abundance of tri-colored bats in Texas culverts. Journal of Mammalogy 100(4):1274–1281. 10.1093/jmammal/gyz099

[CIT0055] Meretsky VJ , Brack V Jr, Carter TC, Clawson R, Currie RR, Hemberger TA, Herzog CJ, Hicks AC, Kath JA, MacGregor JR, et al. 2010. Digital photography improves consistency and accuracy of bat counts in hibernacula. Journal of Wildlife Management 74(1):166–173. 10.2193/2008-306

[CIT0056] Montgomery K. 2006. Variation in temperature with altitude and latitude. Journal of Geography 105(3):133–135. 10.1080/00221340608978675

[CIT0057] Morgan C , Ammerman LK, Demere KD, Doty JB, Nakazawa YJ, Mauldin MR. 2019. Field identification key and guide for bats of the United States of America. Occasional Papers Museum of Texas Tech University 360:1–25. PMC6537616 31148880

[CIT0058] Nagy ZL , Postawa T. 2011. Seasonal and geographical distribution of cave-dwelling bats in Romania: implications for conservation. Animal Conservation 14(1):74–86. 10.1111/j.1469-1795.2010.00392.x 32313439 PMC7159349

[CIT0059] National Wildlife Health Center [NWHC]. 2016. National white-nose syndrome decontamination protocol. Madison (WI, USA): National Wildlife Health Center. [accessed 24 May 2022]. https://www.dfw.state.or.us/wildlife/rehabilitation/docs/National_WNS_Decon_Protocol_041216.pdf

[CIT0060] Perkins JM , Barss JM, Peterson J. 1990. Winter records of bats in Oregon and Washington. Northwestern Naturalist 71(2):59–62. 10.2307/3536594

[CIT0061] Perry RW. 2013. A review of factors affecting cave climates for hibernating bats in temperate North America. Environmental Reviews 21(1):28–39. 10.1139/er-2012-0042

[CIT0062] Piaggio AJ , Valdez EW, Bogan MA, Spicer GS. 2002. Systematics of *Myotis occultus* (Chiroptera: Vespertilionidae) inferred from sequences of two mitochondrial genes. Journal of Mammalogy 83(2):386–395. 10.1644/1545-1542(2002)083<0386:somocv>2.0.co;2

[CIT0063] Piksa K , Nowak J, Żmihorski M, Bogdanowicz W. 2013. Nonlinear distribution pattern of hibernating bats in caves along an elevational gradient in mountain (Carpathians, Southern Poland). PLoS One 8(7):e68066. 10.1371/journal.pone.0068066 23861850 PMC3702566

[CIT0064] R Development Core Team. 2021. R: a language and environment for statistical computing. Version 4.0.2. Vienna (Austria): R Foundation for Statistical Computing. www.R-project.org

[CIT0065] Ramos-H D , Sierra-Durán C, Romero-Ruiz A, Marín G, Cafaggi D, Medellín RA. 2022. Murciélagos hibernantes en el bosque de agua de la Megalópolis de México: Cinco años de experiencia. In: Ávila-Akerberg V, González-Martínez T, editors. Científicos y sociedad en acción por la biodiversidad y la sustentabilidad del bosque de agua de la Megalópolis de México. Mexico City (Mexico): Editorial Instituto de Ciencias Agropecuarias y Rurales, Universidad Autónoma del Estado de México (UAEM); p. 138–144.

[CIT0066] Rivas-Camo NA , Sabido-Villanueva PA, Peralta-Muñoz CR, Medellín RA. 2020. Cuba in Mexico: first record of *Phyllops falcatus* (Gray, 1839) (Chiroptera, Phyllostomidae) for Mexico and other new records of bats from Cozumel, Quintana Roo. ZooKeys 973:153–162. 10.3897/zookeys.973.53185 33110376 PMC7550390

[CIT0067] Rzedowski J. 1978. Vegetación de México. 1st ed. Mexico City (Mexico): Limusa S.A.

[CIT0068] Segura DY. 2010. Registro de murciélagos en “Arcos del Sitio,” municipio de Tepotzotlán, Estado de México [bachelor’s thesis]. [Tlalnepantla (State of Mexico, Mexico)]: Universidad Nacional Autónoma de México.

[CIT0069] Sierra-Durán C. 2020. Potencial de propagación del síndrome de la nariz blanca en México [bachelor’s thesis]. [Bogotá DC (Cundinamarca, Colombia)]: National University of Colombia.

[CIT0070] Sikes RS , The Animal Care and Use Committee of the American Society of Mammalogists. 2016. 2016 Guidelines of the American Society of Mammalogists for the use of wild mammals in research and education. Journal of Mammalogy 97(3):663–688. 10.1093/jmammal/gyw078 29692469 PMC5909806

[CIT0071] Smith LM , Doonan TJ, Sylvia AL, Gore JA. 2021. Characteristics of cave used by wintering bats in a subtropical environment. Journal of Fish and Wildlife Management 12(1):139–150. 10.3996/jfwm-20-078

[CIT0072] Storm JJ , Boyles JG. 2011. Body temperature and body mass of hibernating little brown bats *Myotis lucifugus* in hibernacula affected by white-nose syndrome. Acta Theriologica 56(2):123–127. 10.1007/s13364-010-0018-5

[CIT0073] Vargas JA. 1998. Factores microclimáticos y selección del refugio diurno por murciélagos cavernícolas en Gómez Farías, Tamaulipas [master’s thesis]. [Mexico City (Mexico)]: Universidad Nacional Autónoma de México.

[CIT0074] Villa B. 1966. Los murciélagos de México. Mexico City (Mexico): Universidad Nacional Autónoma de México.

[CIT0075] Webb PI , Speakman JR, Racey PA. 1996. How hot is a hibernaculum? A review of the temperatures at which bats hibernate. Canadian Journal of Zoology 74(4):761–765. 10.1139/z96-087

[CIT0076] Weller TJ , Rodhouse TJ, Neubaum DJ, Ormsbee PC, Dixon RD, Popp DL, Williams JA, Osborn SD, Rogers BW, Beard LO, et al. 2018. A review of bat hibernacula across the western United States: implications for white-nose syndrome surveillance and management. PLoS One 13(10):e0205647. 10.1371/journal.pone.0205647 30379854 PMC6209190

[CIT0077] White-Nose Syndrome Response Team [WNSRT]. 2022. Bats affected by WNS. Madison (WI, USA): White-Nose Syndrome Response Team. [accessed 18 Apr 2022]. https://www.whitenosesyndrome.org/static-page/bats-affected-by-wns

[CIT0078] Whiting JC , Doering B, Wright G, Englestead DK, Frye JA, Stefanic T. 2018. Bat hibernacula in caves of southern Idaho: implications for monitoring and management. Western North American Naturalist 78(2):165–173. 10.3398/064.078.0207

[CIT0079] Woloszyn D , Woloszyn B. 1982. Los mamíferos de la Sierra de la Laguna, Baja California Sur. Mexico City (Mexico): Consejo Nacional de Ciencia y Tecnología.

