# Supplementary material for: Hibernacula of bats in Mexico, the southernmost records of hibernation in North America
Source: J Mammal. 2024 May 3;105(4):823–37. doi: 10.1093/jmammal/gyae027 (PMC11285189; doi:10.1093/jmammal/gyae027)
Supplement: gyae027_suppl_Supplementary_Datas_SD1 [file gyae027_suppl_supplementary_datas_sd1.docx]

**Supplementary Data SD1.—**Adjacent substrate (Tsub) and fur (Tfur) temperatures (in degree Celsius) of torpid vespertilionid bat species reported by similar studies in the United States. Mean values and standard deviations are present.

| Reference | States | Method | Bat species | Tsub (°C) | Tfur (°C) |
| --- | --- | --- | --- | --- | --- |
| Brack (2007) | Ohio | Infrared thermometer | *Eptesicus fuscus* | 9.5 ± 1.5 |  |
|  |  |  | *Myotis lucifugus* | 7.2 ± 2.6 |  |
|  |  |  | *Myotis septentrionalis* | 9.1 ± 0.2 |  |
|  |  |  | *Myotis sodalis* | 8.4 ± 1.7 |  |
|  |  |  | *Perimyotis subflavus* | 9.5 ± 1.9 |  |
| Hopkins et al. (2021) | Michigan, Wisconsin | Infrared thermometer | *Myotis lucifugus* | 8.95 ± 0.02 |  |
| Ingersoll et al. (2010) | Colorado | Infrared thermometer | *Corynorhinus townsendii* | 7.15 |  |
| Langwig et al. (2016)^1^ | New York, Vermont, Massachusetts, Virginia, Illinois | Infrared thermometer | *Myotis septentrionalis* | ≈ 6.7 ± 0.5 |  |
|  |  |  | *Myotis lucifugus* | ≈ 4.2 ± 1 |  |
|  |  |  | *Myotis leibii* | ≈ 0.7 ± 0.6 |  |
|  |  |  | *Myotis sodalis* | ≈ 5 ± 2.2 |  |
|  |  |  | *Perimyotis subflavus* | ≈ 7 ± 0.5 |  |
|  |  |  | *Eptesicus fuscus* | ≈ 2 ± 0.7 |  |
| Meierhofer et al. (2019a) | Texas | Infrared thermometer | *Corynorhinus townsendii* | 11.18 ± 4.36 | 9.3 ± 3.05 |
|  |  |  | *Corynorhinus rafinesquii* | 22.2 ± 4.21 | 20.65 ± 5.85 |
|  |  |  | *Eptesicus fuscus* | 11.73 ± 5.57 | 10.27 ± 3.02 |
|  |  |  | *Myotis auriculus* | 17.36 ± 2.49 | 18.08 ± 2.36 |
|  |  |  | *Myotis velifer* | 14.39 ± 4.87 | 12.41 ± 4.05 |
|  |  |  | *Perimyotis subflavus* | 15.79 ± 3.69 | 15.07 ± 2.87 |
| Smith et al. (2021)^2^ | Florida | Infrared thermometer | *Perimyotis subflavus* | 13 ± 4.4 |  |
|  |  |  | *Myotis austroriparius* | 14.3 ± 4.4 |  |
| Storm and Boyles (2011) | New York | Thermal camera | *Myotis lucifugus* | 4.8 ± 1.5 | 5 ± 1.7 |
|  | Indiana |  | *Myotis sodalis* | 10.1 ± 1.7 | 9.9 ± 1.9 |
| ^1^Data estimated from an original figure. | | | | | |
| ^2^Data expressed from average of wall and ceiling near the highest concentration of bats in each cave. | | | | | |
