# Supplementary material for: Hibernacula of bats in Mexico, the southernmost records of hibernation in North America
Source: J Mammal. 2024 May 3;105(4):823–37. doi: 10.1093/jmammal/gyae027 (PMC11285189; doi:10.1093/jmammal/gyae027)
Supplement: gyae027_suppl_Supplementary_Datas_SD2 [file gyae027_suppl_supplementary_datas_sd2.docx]

**Supplementary Data SD2**.— Extended acknowledgements for contributors of this study.

We are grateful to Leonel Contreras and Marisol Tovar of the Comunidad Agraria La Magdalena Atlitic, Jorge Ayala-Berdon, Kevin Medina, Iván Cabrera, Issachar López, Hugo Vega and Daniela Ceballos of the Universidad Autónoma de Tlaxcala (UATx), Delfino García, Ulises Martínez, Gabriel Martínez and Sara García of the Monitoreo Biológico San Pablo Oztotepec, Tanya González, Ramón Espinasa, and Ana Ibarra of the Universidad Nacional Autónoma de México (UNAM), Heliot Zarza, Jorge Días, Manuel Valdez, Gerardo Ceballos and Jesús Pacheco of the Laboratorio de Ecología y Conservación de Fauna Silvestre (LECFS-UNAM), Víctor Ávila and Fernando Gopar of the Universidad Autónoma del Estado de México (UAEM), Lissette Leyequien, Rita Reyes, Javier Ochoa, Julio Carrera, Candelario Hernández and Claudia Castillo of the Comisión Nacional de Áreas Naturales Protegidas (CONANP), Enrique Pérez and Abdon Martinez of the Ejido Huertecillas, Demetrio Góngora and Adrián Salcedo of the Minera Fresnillo, Elías Ramos of the Expediciones Paquimé, Jonás Delgadillo, Hugo Sotelo, Alejandro Espinosa, Gilberto Martínez and Pablo Medina of the Cementos Mexicanos (CEMEX), Mario Falcón of the Ejidos Jaboncillos, Rafael González and Alexis Ruíz of the Xtreme Mountain Ajusco (XTMA), Rafael Ávila-Flores of the Universidad Juárez Autónoma de Tabasco (UJAT), Miguel León-Galván and Manuel Castillo of the Universidad Autónoma Metropolitana (UAM), Oscar Armas and Concepción Serrano of the Ejido Tecomalucan, Begoña Iñarritu of the Comisión Estatal de Parques Naturales y de la Fauna del Estado de México (CEPANAF), Santiago Niño, Juan Puga, Emanuel Heredia and Gerardo Heredia of the Universidad Autónoma de Tamaulipas (UAT), Abraham Esqueda of the Rancho El Porvenir, Marcial Armendalis and Ambrosio Delgadillo of the Ejido Guadalupe Garzarón, Sergio Marines, Arturo Cruz, Griselda Carmona and Efraín Ramírez of the Profauna A.C., Manuel Mansilla of the Ejido El Recreo, Lorenzo Hinojos of the Rancho El Murciélago, Jonathan Gonzales of the Ejido San Joaquín, Roberto Pedraza of the Grupo Ecológico Sierra Gorda, Ramón León and Jonathan Gómez of the Grupo Alpino Cuitláhuac, Roberto González of the Ejido Tres Álamos, Renzo Romero of the Nómada Aventura, Rosa Morales, Rosario Monero, Víctor Puente and Manuel Romo of the Minera First Majestic, and all volunteers Ángel Campos, Liliana Rosas, Saraith Pérez, Vania Olmos, Abiael Illescas, Samara Pérez, Stephani Rendis, Ángel Torres, Luis de la Fuente, Carolina Palacios, Paula Espino, Daniela Reyes, Ameyalli Marín, Gabriela Reyes, Montserrat Reyes, Samara Kuri, Uriel Fuentes, Mariana González and Sebastián Colotla, who contributed to our project for providing study site permits, improving infrastructure during field work, and for their invaluable field support.
